# Micromechanical resonator with dielectric nonlinearity

**DOI:** 10.1038/s41378-018-0013-6

**Published:** 2018-07-02

**Authors:** Farrukh Mateen, Joseph Boales, Shyamsunder Erramilli, Pritiraj Mohanty

**Affiliations:** 1 Department of Mechanical and Aerospace Engineering, Boston University, 110 Cummington Street, Boston, MA 02215 USA; 2 Department of Physics, Boston University, 590 Commonwealth Avenue, Boston, MA 02215 USA

## Abstract

Nonlinear response of dielectric polarization to electric field in certain media is the foundation of nonlinear optics. Optically, such nonlinearities are observed at high light intensities, achievable by laser, where atomic-scale field strengths exceeding 10^6^–10^8^ V/m can be realized. Nonlinear optics includes a host of fascinating phenomena such as higher harmonic frequency generation, sum and difference frequency generation, four-wave mixing, self-focusing, optical phase conjugation, and optical rectification. Even though nonlinear optics has been studied for more than five decades, such studies in analogous acoustic or microwave frequency ranges are yet to be realized. Here, we demonstrate a nonlinear dielectric resonator composed of a silicon micromechanical resonator with an aluminum nitride piezoelectric layer, a material known to have a nonlinear optical susceptibility. Using a novel multiport approach, we demonstrate second and third-harmonic generation, sum and difference frequency generation, and four-wave mixing. Our demonstration of a nonlinear dielectric resonator opens up unprecedented possibilities for exploring nonlinear dielectric effects in engineered structures with an equally broad range of effects such as those observed in nonlinear optics. Furthermore, integration of a nonlinear dielectric layer on a chip-scale silicon micromechanical resonator offers tantalizing prospects for novel applications, such as ultra high harmonic generation, frequency multipliers, microwave frequency-comb generators, and nonlinear microwave signal processing.

## Micromechanical resonators: Nonlinear acoustic behavior

Coupling of nonlinear polarization in a dielectric medium in a piezoelectric film to mechanical motion leads to a resonator with nonlinear behavior in the acoustic frequency range. Nonlinear dielectric effects are well known to occur at the high optical frequencies typical of lasers, with second harmonic generation (the summation of identical photons to achieve a single photon of twice their energy) being an archetypal signature. Nonlinear dielectric behavior in a low frequency resonator is yet to be achieved however. Now, a team led by Pritiraj Mohanty at Boston University transduce nonlinear behavior in a dielectric medium into a range of mechanical effects—including second harmonic generation—via a piezoelectric material. This demonstration provides a platform for studying nonlinear acoustics, and may offer interesting new device functionality.

## Introduction

The nonlinear relation between electric field and polarization response is at the heart of nonlinear optics^1–4^. In its simplest manifestation, second harmonic generation (SHG) combines two identical photons of frequency *ω*_*1*_ to form a single photon with twice the frequency (*ω*_2_ = 2*ω*_1_). SHG is the most commonly employed commercial technique for frequency doubling of lasers. Similarly, if two photons of dissimilar frequencies (*ω*_1_ and *ω*_2_) are applied, frequency sums and differences can be generated. The next order nonlinear effect, third-harmonic generation (THG), can take an input of three dissimilar frequencies and generate various algebraic combinations of sums and differences from the inputs, in addition to frequency tripling. This is known as four-wave mixing. Other SHG-related fundamental effects include optical rectification, Pockel’s effect, and parametric amplification, and THG-related effects include Kerr nonlinearity and nonlinear Raman scattering^3, 4^.

Analogous nonlinear dielectric effects in the acoustic frequency range could provide similar benefits of harmonic generation, phase conjugation, parametric oscillation, and scattering effects. The simplest approach to achieving a mechanical nonlinear dielectric resonator is to combine nonlinearity in the dielectric polarization with an elastic system, so that an applied electric field can generate nonlinear dielectric response in an appropriate material such as aluminum nitride (AlN)^5–9^. The nonlinear response can be captured in an elastic system capable of transducing the polarization effect into mechanical motion in the acoustic frequency range. One such transduction mechanism is piezoelectric effect, which can transduce an electric field to a mechanical strain. The resulting higher-order components in polarization can manifest (via the piezoelectric effect) as higher-order acoustic frequency components with mechanical vibrations of the resonator. Furthermore, reducing the thickness of the material to the micron scale, and sandwiching the layer between two electrodes, high electric field ranges can be accomplished even with moderate voltages applied across the layer. For instance, 1 volt applied across a thickness of 1 μm generates an electric field of 10^6^ V/m, approaching the regime in which dielectric nonlinearity in the material starts to appear (~10^6^ V/m).

## Materials and methods

Application of an external field in a dielectric material sets up a response field inside the material due to its polarization, reducing the strength of the external field. The polarization field, $\vec P$, is in the same direction as the external field:

1 $$\vec P = \varepsilon _0\chi _e^{\left( 1 \right)}\vec E,$$

where *ε*_0_ is the permittivity of free space and *χ*_*e*_ is the linear (first-order) susceptibility. The total field inside of the material, $\vec E$, is a result of both the free charges given by the displacement field $\vec D$, and the bound charges given by $\vec P$, hence $\vec D = \varepsilon _0\vec E + \vec P$. For a linear dielectric, $\vec D = \varepsilon \vec E$, where the dielectric constant is $\varepsilon = \varepsilon _0(1 + \chi _e^{\left( 1 \right)})$.

As the intensity of the electric field is increased beyond the linear regime, $\vec P$ is given by a Taylor expansion in powers of electric field with coefficients *χ*^(*n*)^ corresponding to the *n*-th order susceptibility:

2 $$\vec P = \varepsilon _0\chi _e^{\left( 1 \right)}\vec E + \varepsilon _0\chi _e^{\left( 2 \right)}\left[ {\vec E} \right]^2 + \varepsilon _0\chi _e^{\left( 3 \right)}\left[ {\vec E} \right]^3 + \ldots$$

Symmetry of the material has a key role in that the second term, responsible for second harmonic generation, is not present in materials, such as silicon, that have centrosymmetric crystalline structures. Such crystals have an indistinguishable point (−*x*, −*y*, −*z*) for every point (*x*, *y*, *z*) within their unit cell. Hence the inversion-symmetric medium is invariant under the parity operator, although it does add a negative sign to both $\vec P \to - \vec P$ and $\vec E \to - \vec E$. This is only possible if the coefficients *χ*^(*n*)^ = 0, for even *n*. However, these coefficients are nonzero in non-centrosymmetric crystals that also exhibit the piezoelectric effect. Thus, second harmonic generation can be produced in piezoelectric materials such as aluminum nitride.

In the presence of high-intensity field in a dielectric medium, the system can be modeled^3, 4^ as an oscillator with a nonlinear restoring force and a driving electric field consisting of two-frequency components *ω*_1_ and *ω*_2_ such that $E\left( t \right) = E_1e^{i\omega _1t} + E_2e^{i\omega _2t} + c.c$. For such a system, the second-order nonlinear polarization is given as

3 $$ \begin{array}{l}P^{(2)} = \varepsilon _0\chi ^{(2)}\left[ {E_1^2e^{ - i2\omega _1t} + \left( {E_1^ \ast } \right)^2e^{i2\omega _1t} + E_2^2e^{ - i2\omega _2t}} \right.\\ + \left( {E_2^ \ast } \right)^2e^{i2\omega _2t} + 2E_1E_2e^{ - i\left( {\omega _1 + \omega _2} \right)t} + 2E_1^ \ast E_2^ \ast e^{i\left( {\omega _1 + \omega _2} \right)t}\\ \left. { + 2E_1E_2^ \ast e^{ - i\left( {\omega _1 - \omega _2} \right)t} + 2E_1^ \ast E_2e^{i\left( {\omega _1 - \omega _2} \right)t} + \varepsilon _0\chi ^{(2)}} \right]\left[ {2E_1E_1^ \ast + 2E_2E_2^ \ast } \right]\end{array}.$$

This expression includes the second harmonic terms *2ω*_*1*_ and *2ω*_*2*_ as well as the sum and difference frequency (three-wave mixing) components *ω*_*1*_ + *ω*_*2*_ and *ω*_*1*_ − *ω*_*2*_. In the case where *ω*_*1*_ and *ω*_*2*_ are equal, the mixing is degenerate. In addition, there is a component corresponding to a steady-state polarization density that creates a static electric field in the material even though a time varying signal is applied—this is the rectification effect^3, 4^. Similarly, when driven by an electric field having three frequency components, $E\left( t \right) = E_1e^{i\omega _1t} + E_2e^{i\omega _2t} + E_3e^{i\omega _3t} + c.c.$, third-order nonlinear polarization reveals multiple four-wave mixing components.

4 $$ \begin{array}{l}P\left( {3\omega _1} \right) = \varepsilon _0\chi ^{(3)}E_1^3;\quad P\left( {3\omega _2} \right) = \varepsilon _0\chi ^{(3)}E_2^3;\quad P\left( {3\omega _3} \right) = \varepsilon _0\chi ^{(3)}E_3^3;\\ \begin{array}{*{20}{c}} {P\left( {\omega _1 + \omega _2 + \omega _3} \right) = 6\varepsilon _0\chi ^{(3)}E_1E_2E_3;} & {P\left( {\omega _1 + \omega _2 - \omega _3} \right) = 6\varepsilon _0\chi ^{(3)}E_1E_2E_3^ \ast ;} \\ {P\left( {\omega _1 - \omega _2 + \omega _3} \right) = 6\varepsilon _0\chi ^{(3)}E_1E_3E_2^ \ast ;} & {P\left( { - \omega _1 + \omega _2 + \omega _3} \right) = 6\varepsilon _0\chi ^{(3)}E_1^ \ast E_2E_3;} \end{array}\end{array}$$

In a nonlinear-optics material, second harmonic generation corresponds to a mechanism where two photons with frequency *ω* are destroyed to produce a single photon with a frequency 2*ω*, thus conserving energy as well as phase; third-harmonic generation corresponds to the creation of a single photon of frequency 3*ω* out of three photons of frequency *ω*. These processes can be extended to the acoustic range where two frequencies can combine to generate a different frequency. For these effects, electric field amplitude needs to be very high, so that the second order and third-order effects (second and third terms in Eq. (2)) are of the same order of magnitude as the first one, $\chi _e^{\left( 1 \right)}\left[ {\vec E} \right]^1\sim \chi _e^{\left( 2 \right)}\left[ {\vec E} \right]^2\sim \chi _e^{\left( 3 \right)}\left[ {\vec E} \right]^3$, or the corresponding field magnitude, $\left[ {\vec E} \right]\sim (\chi _e^{\left( 1 \right)}/\chi _e^{\left( 2 \right)})\sim 1/\chi _e^{\left( 2 \right)}$, as $\chi _e^{\left( 1 \right)}$ is on the order of 1. Hence, in most materials, the dielectric nonlinearity described here requires large electric fields for higher harmonic generation.

This dielectric nonlinearity is fundamentally different from mechanical or elastic nonlinearity which arises due to parametric nonlinearity or nonlinear spring constant^10^, such as in a Duffing oscillator^11, 12^ or in nonlinear damping^13, 14^, or other forms of materials nonlinearity such as piezoelectric nonlinearity involving a nonlinear strain–field relationship. In the context of energy harvesting, nonlinear properties of piezoelectric materials have been explored in piezoelectric constitutive relationship^15^, and in elastic nonlinearity^16^. Nonlinear higher harmonic generation^17^ and four-wave mixing^18^ have been observed in piezoelectric resonators at higher power levels, possibly implying an underlying nonlinear strain–field mechanism or conventional elastic nonlinearity or a combination of mechanisms. The phenomenon is also different from acoustical nonlinear effects studied in microfluidics^19–21^ where motion of particles, microspheres or other objects can be controlled by manipulating fluid motion on microscale.

We use piezoelectric effect, already intrinsic to the chosen material, as a mechanism for transducing polarization effects in the acoustic frequency range. Nonlinear components in polarization as a function of electric field, as described in Eqs. (2–4) can give rise to strain at specific frequencies, determined by the natural resonance frequencies of the resonator. Piezoelectricity consists of two complementary effects—the direct and the inverse effects. The direct effect is the generation of charge polarization as a result of applied stress on the material, while the inverse effect is the generation of strain by applied electric field across the material. The piezoelectric constitutive equations are

5 $$\begin{array}{l}S_{ij} = s_{ijkl}T_{kl} + d^T_{ijn}E_n;\\ D_m = d_{mkl}T_{kl} + \varepsilon _{mn}E_n.\end{array}$$

The first equation represents the inverse piezoelectric effect while the second equation represents the accompanying direct effect. Here *S*_*ij*_ is the rank-2 strain tensor, *s*_*ijkl*_ is the rank-4 compliance tensor, *T*_*kl*_ is the rank-2 stress tensor, *d*_*mkl*_ is the rank-3 piezoelectric coefficient, *ε*_*mn*_ is the rank-2 material permittivity, *E*_*n*_ is the electric field, and *D*_*m*_ is the displacement field. These constitutive equations are only valid in the linear, low-electric-field-intensity regime. For high-intensity electric fields, *D*_*m*_, to the first order, includes both nonlinear terms in the piezoelectric constant (first term in Eq. 5) and the electric field (second term in Eq. 5), as the polarization field *P* is no longer linear. These nonlinear interactions produce multiple frequency components as well, though these nonlinear effects are related predominantly to nonlinearity in the piezoelectric material involving piezoelectric coefficients with higher-order terms. The dielectric nonlinear effects, described in Eqs. (2–4) require a material with non-centrosymmetry, but not all non-centrosymmetric materials are piezoelectric. Absence of center of symmetry is a necessary but not a sufficient requirement for a material to exhibit an appreciable piezoelectric effect. The nonlinear effects arising out of nonlinear field–polarization relationship at large electric fields can be observed even when the strain–field relationship is linear.

## Results

**Fig. 1:**
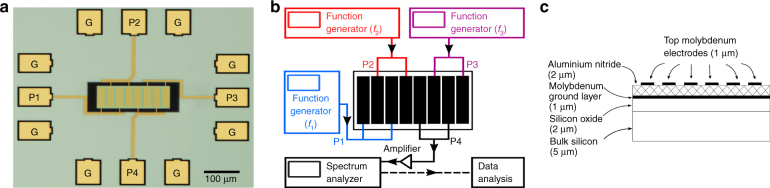
Device micrograph and schematic circuit diagram and materials layer stack. **a** Micrograph at ×20 magnification of the four-port micromechanical resonator. The tabs marked as P1, P2, P3 and P4 can be used symmetrically as the inputs and outputs, while the tabs marked with G are the common ground for the device. Throughout this paper, inputs with different frequencies are applied to Ports 1, 2 and 3 (P1, P2, and P3, respectively) while the output is measured at port 4 (P4). The central rectangular plate-type piezoelectric element of the resonator measures 100 by 245 μm and is straddled by eight (molybdenum) interdigitated transducers (IDTs). Two of these transducers are connected to each of the ports by thin molybdenum connects. **b** Circuit diagram for the measurement setup is presented. A schematic of the resonator is presented in the center while the ports 1 to 3 (P1 to P3) are presented in blue red and purple, respectively. Port 4 (P4) is part of the output circuit and is presented in black. The three function generators provide the three input frequency signals (*f*_1_, *f*_2_, and *f*_3_, respectively) to ports P1 to P3. The output at port 4 (P4) is fed through a low-noise voltage amplifier before being collected by a signal analyzer. **c** The stack of materials in the suspended part of the resonator consist, from bottom to top, a silicon layer (5 μm thick), an oxide layer (2 μm thick) and the first electrode layer (1 μm thick), aluminum nitride layer (2 μm thick) and the second electrode layer (1 μm thick)

We use a micron-sized piezoelectric resonator, shown in Fig. 1a, consisting of an aluminum nitride (AlN) piezoelectric layer sandwiched between two layers of metallic electrodes which are deposited on a suspended silicon resonator. AlN has a non-centrosymmetric crystalline structure, enabling both second and third-harmonic generation, whereas the underlying silicon layer with centrosymmetric crystal structure may contribute to only third-harmonic generation. To detect ultra-small signals arising from nonlinear interactions, we employ a multiport design to actuate and detect the resonator’s response. With multiple ports, the resonator can be operated in a common-mode configuration to increase the signal-to-noise ratio and avoid electrical mixing. Even though the nonlinear medium material generates higher-order nonlinear polarization at all frequencies, the resonator displays responses corresponding to its resonance frequencies where on-resonance signals are enhanced by their respective quality factor.

The micrograph in Fig. 1a shows the plate-type four-port piezoelectric resonator device, about 250 by 100 μm. The resonator consists of two layers of molybdenum (each 1 μm thick) which sandwich the aluminum nitride (2 μm thick) piezoelectric layer, followed by layers of silicon oxide and bulk structural silicon layers. In our experiment, the highest power of 5 dBm (or 1.125 V peak-to-peak) across a 2-μm layer of aluminum nitride corresponds to a maximum attainable electric field of 0.563 × 10^6^ V/m peak-to-peak. The suspended resonator is connected to the substrate by thin anchors. The top molybdenum layer is deposited in the form of four sets of interdigitated transducer (IDT) electrodes; each set is comprised of two electrodes and connected to one of the four square molybdenum tabs on all four sides of the device. These ports (labeled P1 through P4) are the four ports of the resonator device, used symmetrically to provide radio frequency (RF) inputs to or receive output signals from the device. In this study, output signals are measured at P4 while inputs are provided at P1, P2, and P3, as needed. The tabs labeled “G” are used for device grounding. As shown in Fig. 1b, function generators were used to provide either two or three RF inputs (as required) at ports 1 through 3 while the output was measured by a spectrum analyzer connected to port 4 (P4) after being amplified by a low-noise amplifier. Figure 1c shows the layers of materials in the suspended part of the resonator stack, where the AlN layer is sandwiched between two metal electrodes.

**Fig. 2:**
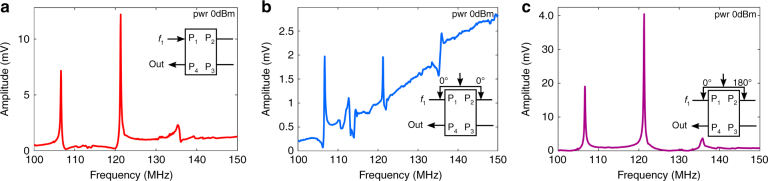
Comparison of common mode and differential mode inputs. All measurements are made at 0 dBm input signal power. Input signals swept between 100 and 150 MHz are applied to the respective ports as indicated in each inset (accompanying the plots). **a** This figure depicts the resonator response in a frequency span of 100 to 150 MHz. The inset shows that Port 1 (P1) is used to provide the excitation while a response is collected from Port 4 (P4). Ports 2 and 3 are not employed. The response shows two major resonance peaks at 106.69 and 121.3 MHz. However, there is some noise in the observed signal. **b** The input is split equally between Ports 1 and 2 (P1 and P2, respectively). The noise in the signal increases considerably and the major resonance peaks at 106.69 and 121.3 MHz reduce in magnitude, considerably. As the spacing between the electrodes is quarter of a wavelength the net two signals interfere destructively causing the reduction in magnitude. The rise in noise offset may be due to stray capacitances that the circuit becomes more prone too as the signal to noise ratio decreases. **c** Differential inputs are applied to Ports 1 and 2 (P1 and P2, respectively). The inset shows that the inputs applied are 180° out of phase. Owing to this, the observed signal has a higher signal-to-noise ratio. The differential input scheme cancels out the common input noise and both Ports 1 and 2 to provide a cleaner—less noisy—response

We start with a frequency sweep between 100 and 150 MHz at P1 while recording the response at P4. The response, shown in Fig. 2a, shows two resonance peaks, one at 106.69 MHz and the other at 121.3 MHz. We next provide identical, in-phase signals at P1 and P2. The results, shown in Fig. 2b, show a reduction of the signal and increase in noise. The largest resonance peak (located at 121.3 MHz) is also reduced in amplitude. Finally, we provide identical, but 180° out-of-phase, signals to P1 and P2. The common-mode rejection response is shown in Fig. 2c, which depicts an increase in the signal-to-noise ratio in comparison to the response shown in Fig. 2a and b. In fact, the application of the same signal with a 180° phase difference at two consecutive ports results in the canceling of the common-mode noise signals, which are applied equally to both parts of the inputs. This scheme can be locally employed to provide a cleaner, less noisy response. For nonlinear frequency-mixing measurements, it also allows application of different frequency signals at different ports without any electrical signal mixing.

**Fig. 3:**
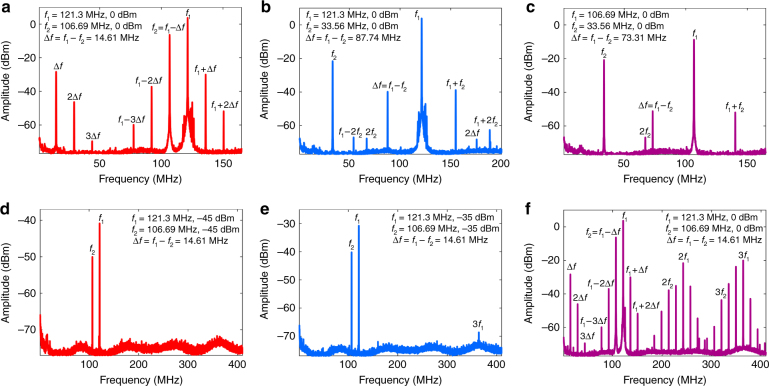
Degenerate four-wave mixing and harmonic generation. **a**–**c** First harmonic excitation of degenerate four-wave mixing (using two frequencies only). The input frequencies, shown in inset, are applied at 0 dBm to Ports 1 and 2 (P1 and P2, respectively) while an output is generated at Port 4 (P4). Port 3 (P3) is not used. In all cases multiples of the input frequencies, along with the sums and differences of the two frequencies, are observed. **d**–**f** Power dependence for the two input frequencies, 121.3 and 106.69 MHz, applied to ports 1 and 2 (P1 and P2, respectively) is shown. **d** When both frequencies are applied at −45 dBm (or below), only the first harmonics are observed. **e** −35 dBm is found to be the threshold voltage between the second harmonic generation and third-harmonic generation. At or below this power only the third-harmonic is visible which is generated by silicon. **f** At 0 dBm input power, complete four-wave mixing is observed and the first three harmonics are visible. The finger like signals on either side of the multiples of frequency *f*_1_ (121.3 MHz) are its sums and differences. The same for the first harmonic are marked, however due to scarcity of space the sums and differences for the second and third harmonics are not marked

The resonator has large resonance peaks at the frequencies of 121.3, 106.69, and 33.56 MHz. We next employ three-wave mixing scheme for second harmonic generation using two frequencies (*f*_1_ and *f*_2_) applied to P1 and P2 at 0 dBm while an output is recorded at P4. The response plots for these frequency pairs *f*_1_ = 121.3 MHz and *f*_2_ = 106.69 MHz, *f*_1_ = 121.3 MHz and *f*_2_ = 33.56 MHz, and *f*_1_ = 106.69 MHz and *f*_2_ = 33.56 MHz are shown in Fig. 3a–c, respectively. The algebraic sum and difference components can be seen in each of the plots.

Next we explore the threshold input signal power for the two frequencies *f*_1_ = 121.3 MHz, *f*_2_ = 106.69 MHz, applied at P1 and P2, at which the first component of harmonic generation appears. Figure 3d, shows the linear response containing only the two resonance peaks at input signal power of −45 dBm. Next the power is increased, and, as depicted in Fig. 3e, we find that the third-harmonic component of 3*f*_1_ appears at −35 dBm. Silicon, which forms the structural base of the resonator, is a dielectric however since it has an inversion-symmetric crystalline structure, so it produces the third-harmonic component. As the power is increased further to 0 dBm, we observe a linear response along with second and third-harmonic components, as depicted in Fig. 3f. Here, the second harmonic component is produced entirely due to the nonlinearity inherent in the aluminum nitride layer. As discussed earlier, in an acoustic nonlinear medium such as the hybrid micron-sized resonator, three-wave mixing produces a complete set of frequency components.

**Fig. 4:**
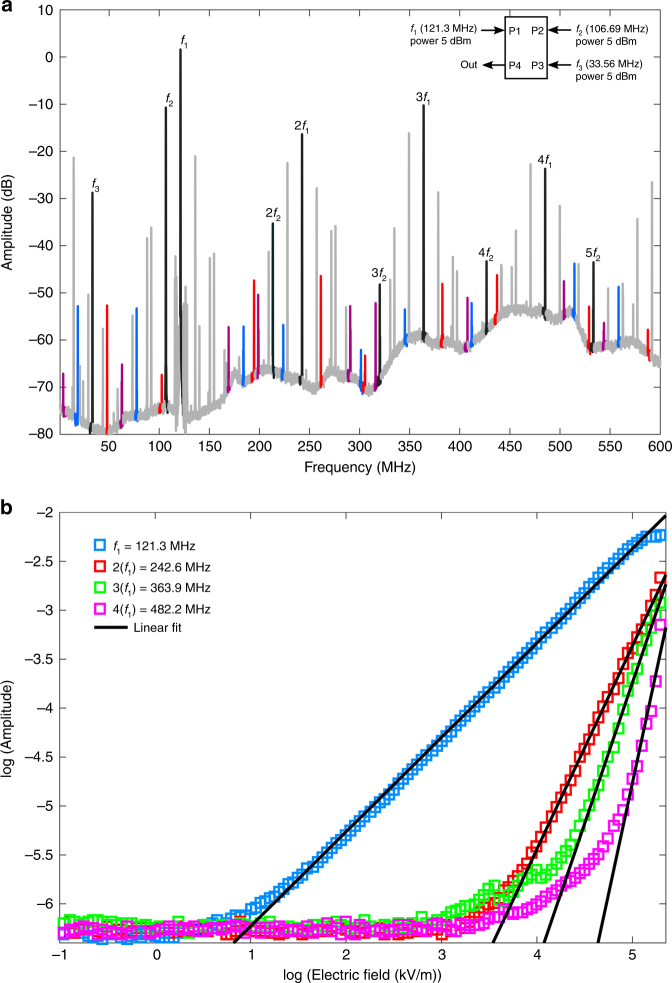
Non-degenerate four-wave mixing. **a** Four-wave mixing, where frequencies *f*_1_ = 121.3 MHz, *f*_2_ = 106.69 MHz, *f*_3_ = 33.56 MHz, are applied at Ports 1, 2, and 3 (P1, P2, and P3, respectively), at 5 dBm, and an output is observed at Port 4 (P4). In colors of purple, blue, and red, the peaks for the four-wave mixed frequencies are highlighted. The applied frequencies *f*_1_, *f*_2_, and *f*_3_ and their multiples as they appear are shown in black. The grayed out peaks are the degenerate algebraic compositions of any two of the three frequencies. The plot depicts the span covering the first four harmonics. It can be seen from the marked peaks and the inset (top right corner) that complete four-wave mixing is observed. The signal due to common-mode inputs does depict considerable noise above the second harmonic. **b** Effect of applied electric field on the peak height of the first four harmonics of the 121.3 MHz mode is shown on a log-log plot. The four harmonics display the expected power law dependence of the polarization on the applied field

Next, we apply three frequency signals, *f*_1_ = 121.3 MHz at P1, *f*_2_ = 106.69 MHz at P2, and *f*_3_ = 33.56 MHz at P3, each at 5 dBm. We observe complete four-wave mixing. As shown in Fig. 4, linear response along with second, third and fourth harmonics are visible. Here, for easier viewing, signals related to components consisting of various positive combinations of two out of the three applied frequencies have been grayed out, while those representing only four-wave mixing are colored (red, blue, or purple). The applied input signal peaks and their multiples are shown in black. Each signal corresponds to within two decimal places of the actual analytical calculation. It is noteworthy that increasing the power of the applied signals further increases the amplitudes of the resultant components and also adds higher harmonics.

We further investigate the effect of the applied electric field on the peak height of the first, second, third and fourth harmonic for the 121.3 MHz mode. We apply a signal at frequency (*f*_1_) at P1 and measure the response at P4. The log-log plot of this dependence is shown in Fig. 4b. The four data sets, corresponding to the four harmonics, are each overlaid with a linear fit. As expected from the field dependence of polarization, $P\sim {\it{\epsilon }}_0\left( {\chi ^{\left( 1 \right)}} \right.E + \chi ^{\left( 2 \right)}E^2 + \chi ^{\left( 3 \right)}E^3 + \chi ^{\left( 4 \right)}E^4 + \ldots$ the first four harmonic peaks scale with power law dependence with exponents approximately equal to 1, 2, 3, and 4, respectively. First-order susceptibility *χ*^(1)^ is a property of the material. For AlN, *χ*^(1)^ is 4.8 along the *c*-axis^16^. Starting with a fixed electric field value of 100 kV/m, we estimate second-order susceptibility *χ*^(2)^ as 4.3 × 10^−6^ m/V, third-order susceptibility *χ*^(3)^ as 21.5 × 10^−22^ m/V and fourth-order susceptibility *χ*^(4)^ as 20.0 × 10^−18^ m/V, at a frequency of 121.3 MHz at room temperature. Further measurements of higher harmonics for two additional mode frequencies of 106 and 33 MHz display similar trends.

## Conclusion

Using a novel multiport resonator, we show that a common-mode rejection scheme can greatly enhance the signal-to-noise ratio by applying the same signal to the two inputs 180° out-of-phase with respect to each other of a four-port piezoelectric resonator. We demonstrate realization of a micromechanical nonlinear dielectric resonator and display the entire suite of three-wave and four-wave mixing components. In addition to enabling the study of a broad range of novel nonlinear acoustics effects, our demonstration can enable unprecedented applications in micron-scale devices, including frequency generators and multipliers, chip-scale frequency-comb generators, and other nonlinear signal processing devices. In particular, electric field magnitude can be increased by orders of magnitude by a thinner layer of non-centrosymmetric material to gain access to higher-order effects. Similar to nonlinear optics systems, higher harmonic generation can be of very high order, as high as several hundreds (or even several thousands^22^). A chip-scale resonator with fundamental frequencies in the 100 MHz range, capable of being used as a frequency source in the 1–10 GHz frequency band, is exciting. Furthermore, CMOS-compatible process for manufacturing of such devices can accelerate real-world use of nonlinear acoustics with a scope comparable to—and perhaps beyond—that of nonlinear optics.
